# Aberrant DNA Methylation in ES Cells

**DOI:** 10.1371/journal.pone.0096090

**Published:** 2014-05-22

**Authors:** Guy Ludwig, Deborah Nejman, Merav Hecht, Shari Orlanski, Monther Abu-Remaileh, Ofra Yanuka, Oded Sandler, Amichai Marx, Douglas Roberts, Nissim Benvenisty, Yehudit Bergman, Monica Mendelsohn, Howard Cedar

**Affiliations:** 1 Department of Developmental Biology and Cancer Research, The Hebrew University–Hadassah Medical School, Jerusalem, Israel; 2 Department of Genetics, Silberman Institute of Life Sciences, The Hebrew University, Jerusalem, Israel; 3 Department of Biochemistry and Molecular Biology, The Hebrew University–Hadassah Medical School, Jerusalem, Israel; 4 Agilent Technologies, Inc., Agilent Laboratories, Santa Clara, California, United States of America; 5 Department of Biochemistry and Molecular Biophysics, Columbia College of Physicians and Surgeons, New York, New York, United States of America; The Babraham Institute, United Kingdom

## Abstract

Both mouse and human embryonic stem cells can be differentiated in vitro to produce a variety of somatic cell types. Using a new developmental tracing approach, we show that these cells are subject to massive aberrant CpG island de novo methylation that is exacerbated by differentiation in vitro. Bioinformatics analysis indicates that there are two distinct forms of abnormal de novo methylation, global as opposed to targeted, and in each case the resulting pattern is determined by molecular rules correlated with local pre-existing histone modification profiles. Since much of the abnormal methylation generated in vitro appears to be stably maintained, this modification may inhibit normal differentiation and could predispose to cancer if cells are used for replacement therapy. Excess CpG island methylation is also observed in normal placenta, suggesting that this process may be governed by an inherent program.

## Introduction

Both mouse and human embryonic stem cells can be derived from early pre-implantation embryos and grown indefinitely in culture. These cells maintain their pluripotency as indicated by the observation at least in mouse that they can generate a full organism [1], and both human and mouse ES cells can be differentiated in vitro to a large variety of different cell types [2], [3]. These studies suggest that embryonic stem cells harbor an epigenetic profile similar to that of the embryo itself and that this pattern has within it the plasticity to generate differentiated cell types.

One of the major epigenetic marks of the genome is DNA methylation. In the early pre-implantation embryo, DNA methyl groups derived from the gametes are largely erased and a new bimodal pattern is re-established in every individual at about the time of implantation [4], [5]. This basal pattern is generated by a wave of indiscriminate de novo methylation in conjunction with a mechanism for protecting CpG island-like sequences on the basis of local cis acting elements [6], [7]. Following this step, differentiated cells lose the ability to carry out global de novo methylation [8], but the basic pattern is none-the-less maintained through every cell division by Dnmt1, which recognizes hemimethylated sites generated at the replication fork [9]. As development proceeds, each individual cell type can then undergo additional de novo methylation [10], [11] or demethylation [4] events in a gene-specific manner.

Although mouse embryonic stem cells are derived from the ICM, genome-wide analysis indicates that, unlike the blastocyst, they are methylated in a manner similar to somatic cells, suggesting that from the epigenetic standpoint, they actually behave like cells at the time of implantation [4]. This observation is substantiated by the fact that ES cells retain the ability to carry out global de novo methylation and are capable of distinguishing and protecting CpG islands from this process [6], [7]. The same is probably true of human ES cells, as well. Upon differentiation in vitro, ES cells appear to undergo appropriate changes in DNA methylation, as indicated, for example, by the well-documented de novo methylation that takes place on pluripotency genes, such as Oct-3/4 and Nanog [12]. Nonetheless, several studies indicate that differentiation in vitro may also be accompanied by aberrant de novo methylation at CpG islands [13]. Since this type of modification is thought to be irreversible in the post-implantation embryo in vivo, it is likely that these abnormal events could adversely affect the quality of differentiated tissues derived from ES cells in culture.

In order to study this problem, we used developmental principles to generate a new approach for assessing what should be considered a normal methylation pattern in ES cells. On the basis of this in vivo perspective, we found that both mouse and human ES cells growing in vitro have aberrant DNA methylation that could have physiological effects on their ability to undergo proper differentiation.

## Materials and Methods

### Differentiation of ES cells

Mouse endoderm and mesoderm were differentiated from ES-GscgfpSox17huCD25 cells originally derived from line EB5 by sequence targeting [14]. Briefly, ES cells were plated on collagen-coated culture dishes and differentiated in a serum-free culture medium with 10 ng/ml human Activin A. Cells were collected after 6 days and subjected to FAC sorting to obtain definitive endoderm (Gsc^+^Sox17^+^ECD^+^) or mesoderm (Gsc^+^Sox17^−^ECD^−^). Embryoid bodies (EBs) were formed from ES cells (TT2) [15] diluted and grown in medium without LIF by the hanging drop method. After 2 days, aggregates were pooled and cultured in suspension for 4 additional days in bacterial Petri-dishes. Mouse teratomas were generated by resuspension of ES cells (D3,C4) into PBS-Basement membrane matrix (1/1) and subcutaneous injection into NOD-SCID mice. Animals were sacrificed 2 weeks after injection. ES cells (J1) [16] were treated with 1 µM retinoic acid (RA) for 8 days [17] in order to induce a neuro-ectodermal population.

Human ES cells (CSES2) [18] were grown on feeder layer and induced to EBs as described previously [19]. Briefly, cells were transferred using trypsin/EDTA to plastic Petri dishes to allow their aggregation and prevent adherence to the plate. In order to induce neuro-ectodermal differentiation, 1 µM RA was added to monolayer ES cells grown on gelatin-coated plates and cells were harvested after 10 days. Undifferentiated human ES cells (I6, H13) [10] were sorted using the SSEA3 marker prior to mDIP analysis.

### mDIP microarrays

Human fetal (20–25 weeks) DNA samples were purchased from Biochain. All mouse DNAs were extracted from C57Bl/6 and mDIP was performed as described previously [10]. Human (10 µg) or mouse (40 µg) genomic DNA was sonicated to an average fragment size of 300–1000 bp, precipitated with 400 mM NaCl, 2 volumes of ethanol and 1 µl glycogen. 1.5 µg were set aside as the Input fraction. DNA was denatured and anti-5-methylcytidine monoclonal antibody (10 µl for 5 µg) [20], [21] was added and incubated on a rotator at 4°C overnight. 40 µl Dynabeads (Sheep anti-Mouse IgG) were prewashed with 0.1% BSA/PBS and added to the DNA. The DNA was then washed 3 times and Ab-bound DNA resuspended and extracted with proteinase K, phenol-chloroform and ethanol precipitation. Purified DNA was checked for enrichment (Bound/Input) using Real time PCR on specific gene regions known to be methylated.

The Input and Bound DNAs were labeled and hybridized on mouse (105 K) or human (244 K) CpG island microarrays (Agilent Technologies, http://www.genomics.agilent.com) as described previously [10]. We used feature extraction software (Agilent) to obtain background-subtracted intensity values for the two fluorescence dyes on each individual array feature and for carrying out linear normalization and calculation of the log ratio (Cy5/Cy3). Data was analyzed as previously described [10]. Briefly, probe log ratio signals were transformed into Z-scores according to their Tm and an Island Methylation Score (IMS) was then calculated by averaging the island corresponding probes' Z-scores. Data for both mouse (GSE54664) and human (GSE24270) analyses can be accessed from the GEO-NCBI database repository.

### Data analysis

Previous published work on undifferentiated and differentiated ES cells and in vitro differentiation was analyzed by downloading data from the appropriate GEO datasets. We used genomic bisulfite data (GSE11034) for mouse DNA [5], [13]. Methylation percent was calculated for every CpG that had a minimum of 5 reads and these were then aligned in order to generate an average methylation value for each CpG island (as defined by UCSC). Islands with data for less than three CpGs were not included. H3K27 tri-methylation (GSE12241) from mouse ES cells [22]. The average density score per CpG island was calculated with background taken as the average score for all CpG islands. Whole-genome human DNA methylation was downloaded from the roadmap epigenomics project (http://www.roadmapepigenomics.org/) as determined by Reduced representation bisulfite sequencing (RRBS).

Previous published work on de-novo methylation in cancer was analyzed by downloading data from The Cancer Genome Atlas (TCGA - https://tcga-data.nci.nih.gov/tcga/) and average methylation values were calculated for every CpG island. Human ChIP-seq data for H3K27 and H3K4 tri-methylation was obtained from the NIH Roadmap Epigenomics Mapping Consortium. We compared 200 CpG islands deemed abnormally methylated in serum-grown ES cells and found them to be about 10% more methylated than the levels seen when cultured in 2i conditions using previously published data (GSE42929) [29].

### Statistical analysis

The significance (P value) of difference between background-island DNA methylation patterns in somatic tissues as opposed to differentiated and undifferentiated ES cells was determined by a two-tailed, non-paired, equal-variance T-test. The significance of H3K27me3 enrichment on target CpG islands as well as comparison between CpG island sets was calculated using the hypergeometric test [23], [24].

### Bisulfite analysis

Single island reads were obtained by Bisulfite conversion of genomic DNA that was carried out using the EZ DNA Methylation-Direct Kit (Zymo Research) according to the manufacturer's instructions. PCR primers were designed using Methyl Primer Express® Software v1.0 (https://www2.appliedbiosystems.com). Barcodes and adaptors were added to the primers and deep-sequenced using the Ion Torrent (Life Technologies).

### Ethics Statement

This study was carried out in strict accordance with the recommendations in the Guide for the Care and Use of Laboratory Animals of the National Institutes of Health. The protocol was approved by the Institutional Animal Care and Use Committee of the Hebrew University (NIH approval number: OPRR-A01-5011).

## Results

Mouse ES cells were grown in culture and induced to differentiate using a variety of commonly-employed strategies (Materials and Methods). Embryoid bodies were formed by aggregation. We treated cells with retinoic acid to induce a neuro-ectodermal population and also purified definitive endoderm or mesoderm from monolayer cultures exposed to Activin induction [25]. DNA was then isolated and subjected to mDIP microarray analysis which measures the average methylation level of all CpG moieties in every island of the genome [10], and the results analysed by comparison to DNA samples from a large panel of normal adult tissues.

**Figure 1:**
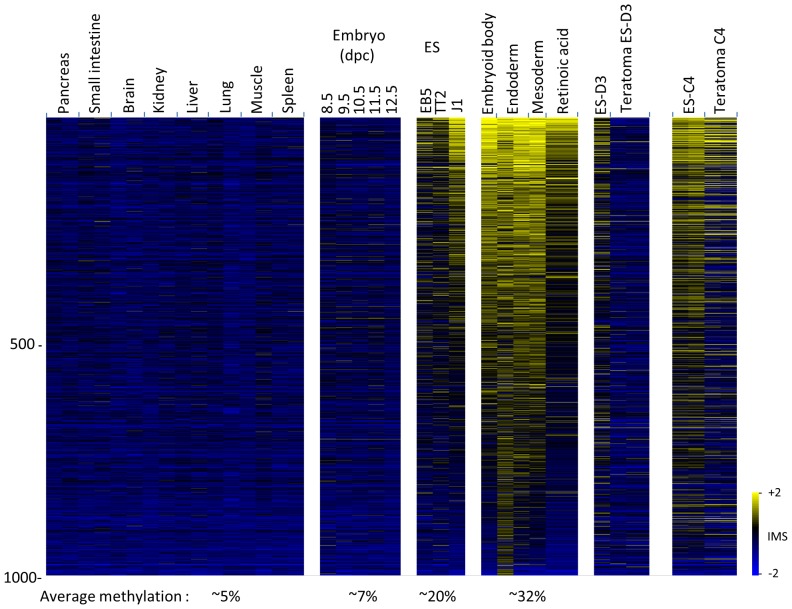
Excess methylation in differentiated mouse ES cells. DNA from normal mouse tissues, undifferentiated and differentiated ES cells, post implantation embryos and teratomas were subject to mDIP microarray analysis. Heat map of 1,000(IMS>0.75) in at least one of the ES cell types out of 9,500 CpG islands constitutively unmethylated (IMS<0) in all tissue samples (see Materials and Methods). A number of different ES cell lines were used in this study. Embryoid bodies and retinoic acid treated cells were derived from TT2, while endoderm and mesoderm were derived from EB5. An estimate [10] for the average percent of methylation in fetal tissues as compared to in vitro differentiated cells is also shown.

We identified about 9,500 CpG islands constitutively unmethylated in every cell type (Materials and Methods). This pattern reflects events that occur at the time of implantation when the entire genome becomes de novo methylated while almost all CpG islands are protected. This basic bimodal profile represents a “ground state” which is then maintained during every cell division throughout development [26]. Strikingly, hundreds of these CpG islands were found to be highly modified (Z-score>0.75) in ES cells differentiated in vitro (Fig. 1). Since most of these islands do not appear to be substantially methylated anywhere in the organism (Z-score<0), it is likely that this in-vitro modification represents some form of artifact that does not reflect the normal epigenetic status at these early stages of embryogenesis. Further support for this idea was obtained by carrying out mDIP on DNA from early mouse embryos (8.5–12.5 dpc) and by comparing to data from near-implantation embryos (Fig. S1a in File S1) which show that the real in vivo level of methylation at these target CpG islands is indeed very low.

This phenomenon of aberrant methylation is not unique to the specific differentiation experiments carried out in our laboratory. Using partial genome-wide bisulfite sequencing, it has already been shown that ES cells differentiated in vitro to neural precursors (NPCs) undergo de novo methylation (>80%) at over 300 CpG island promoters, even though almost all of these same sequences were completely unmethylated in corresponding neural embryonic cells representing this same approximate stage of development [13]. In order to validate this finding using our developmental approach, we carried out additional bioinformatics analysis on this same database. We were able to discern over 2,000 CpG islands aberrantly methylated (average>50%) following differentiation and thousands of additional sequences show methylation levels significantly higher than their normal values in a variety of somatic cell types (Fig. S1a in File S1), generating a pattern very similar to that determined by mDIP analysis (P<10^−132^) (Materials and Methods).

Since the excess modification observed in differentiated ES cells was originally detected in comparison to normal tissue DNA, we next examined the methylation state of these same CpG islands in undifferentiated mouse ES cells. While the same target sequences appear to be relatively unmethylated in these undifferentiated cells, surprisingly, they were found to be much more methylated than the normal population of CpG islands in the cell (Fig. S1b & S1c in File S1), and there is some evidence that this may increase as a function of time in culture [13], [27], [28]. It is unlikely that this excess methylation is representative of the implantation embryo in vivo. Indeed, these target sites were actually found to be unmethylated in DNA from 6.5 and 7.5 d embryos (Fig. S1a in File S1). It should be noted that by growing ES cells in 2i medium, it is possible to lower DNA methylation to levels similar to those seen at the ICM stage [29], [30], [31], and in this way remove most of the excess CpG island methylation (Materials and Methods). It is unlikely, however, that these ground-state cells would generate differentiated derivatives with normal methylation (see [31]) since they would still be required to pass through a more advanced ES cell state prior to differentiation in vitro.

**Figure 2:**
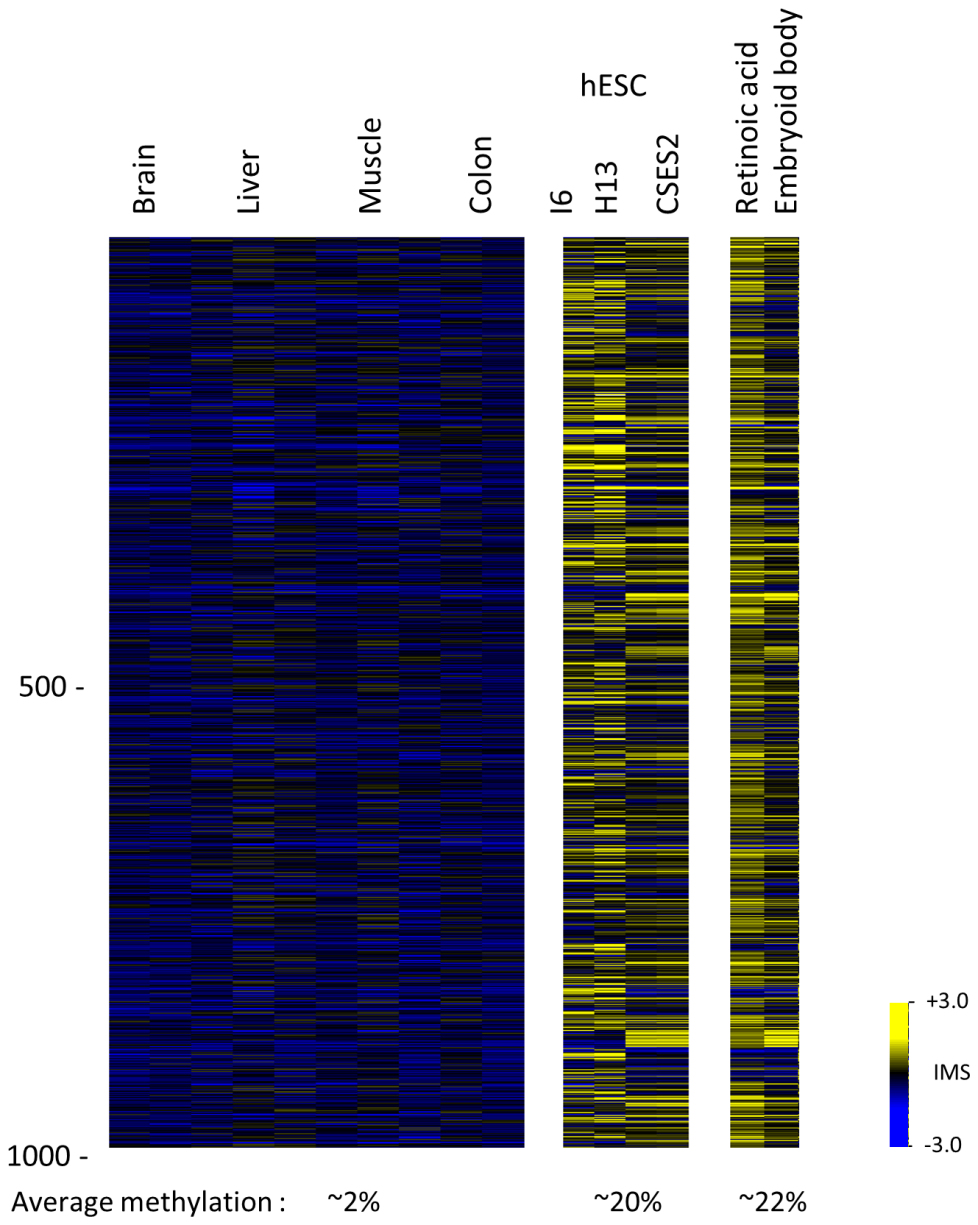
Excess methylation in human ES cells. DNA from normal human fetal tissues, undifferentiated and in vitro differentiated ES cells were subject to mDIP microarray analysis. Heat map shows 950(IMS>0.75) in at least one of the differentiated ES cell types out of 13,000 CpG islands constitutively unmethylated (IMS<0) in fetal tissue samples. Retinoic acid treated cells and embryoid bodies were derived from CSES2. An estimate [10] for the average percent methylation in fetal tissues as compared to in vitro differentiated cells is also shown.

We next asked whether aberrant de novo methylation also occurs in human ES cells. To this end, we used mDIP to analyze CpG island methylation patterns in a number of different undifferentiated ES cell lines and in cell cultures induced to differentiate by several alternative techniques. Once again, the data was compared to normal DNA extracted from human embryo tissues. As noted previously [10], the human genome has about 13,000 constitutively unmethylated CpG islands, and differentiated ES cells demonstrated strong methylation in almost 1,000 of these sequences (Fig. 2). We then analyzed published methylation data on human ES cells that was obtained using high resolution RRBS (www.roadmapepigenomics.org), exclusively focusing on CpG islands that are unmethylated in a wide range of different tissue cell types. Here too, differentiated ES cells were found to have a large number of aberrantly methylated sequences similar to those identified by mDIP analysis (P<10^−124^) and the same was true for induced pluripotent stem (iPS) cells, as well (Fig. S2 in File S1).

As is the case with mouse ES cells, the methylation targets detected in differentiated cells were found to be modified in several human ES cell lines even prior to differentiation, with the level of modification being quite high, and in some cases very similar to that seen in differentiated cells derived from the same lines (Fig. 2). This over-modification was observed even though the human ES cells used for this analysis were actually pre-sorted specifically to select for the undifferentiated phenotype (Materials and Methods). Furthermore, by examining published data from other laboratories, it appears that this same phenomenon is typical of many other human ES cell lines, as well (Fig. S2 in File S1).

It should be noted that while several previous studies identified aberrant de novo methylation during mouse [13], [32] or human [28] ES-cell differentiation in vitro, the normal-tissue tracing strategy that we have introduced in order to understand these events from a developmental perspective, has revealed a much larger range of abnormally methylated CpG islands. This is especially true for the DNA modification seen in undifferentiated human ES cells which lack a normal tissue control and would never have been detected without having the benefit of the developmentally-projected pattern derived from somatic tissues. Taken together, our studies indicate, for the first time, that the generation of excess methylation in culture represents an intrinsic process that actually begins in undifferentiated ES cells and may then be exacerbated by a wide variety of in vitro differentiation techniques.

**Figure 3:**
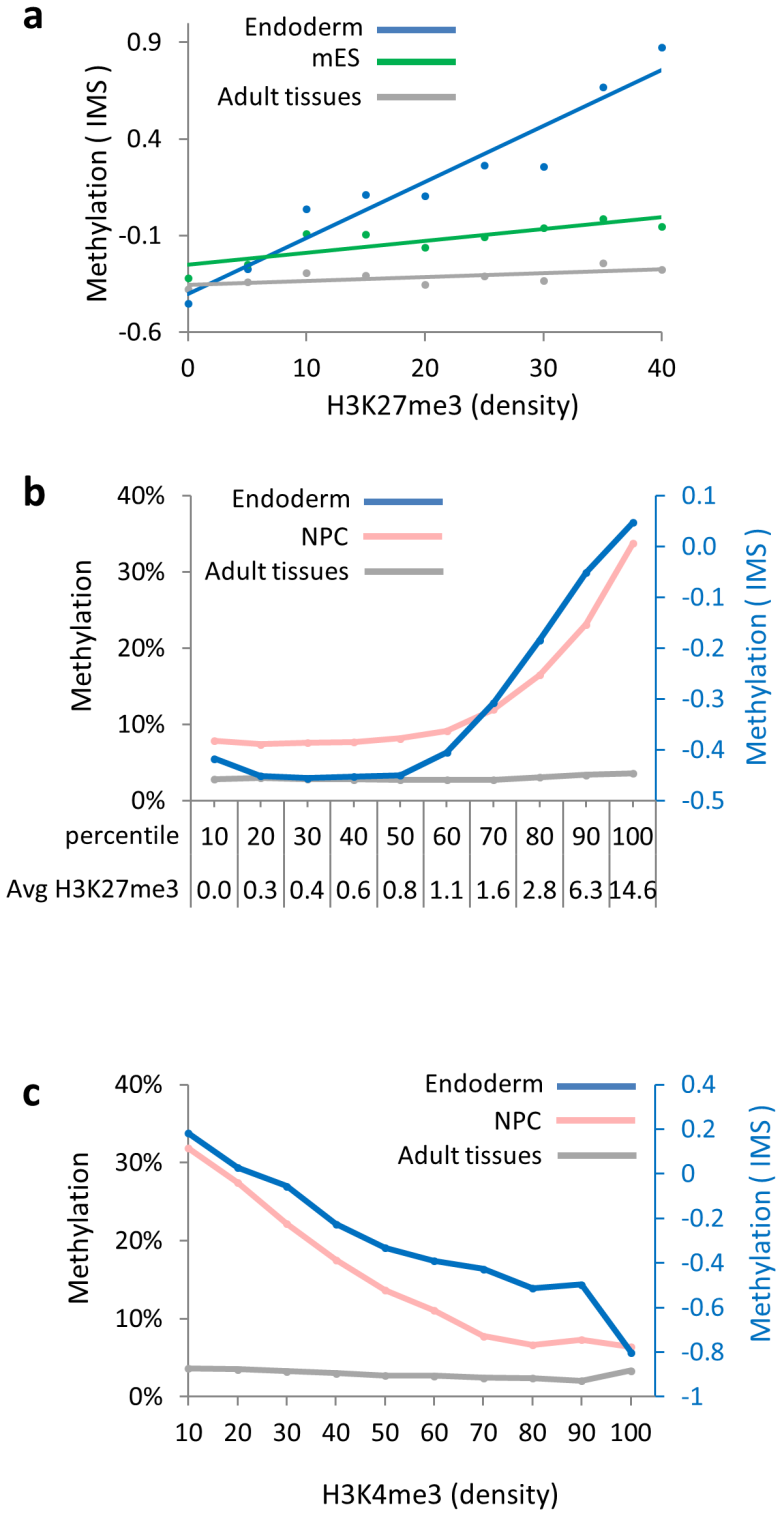
De novo methylation is proportional to H3K27me3 concentration. **a**. IMS (Fig. 1) of all 9,500 constitutively unmethylated CpG islands in undifferentiated, endoderm-differentiated ES cells and adult tissue DNA graphed as a function of H3K27me3 density [22]. Each point represents the average IMS within a 5 unit span. **b**. Average methylation levels of all constitutively unmethylated CpG islands in endoderm (IMS), NPCs (%) and adult tissue (%) were graphed against H3K27me3 density partitioned into ten bins. The X-axis also shows average H3K27me3 levels in each percentile. **c**. Methylation levels in adult tissue, ES differentiated into endoderm and NPCs of all constitutively unmethylated CpG islands with above background concentrations (>2) of H3K27me3 graphed against their H3K4me3 density in ES.

During normal development, almost all post-implantation de novo methylation takes place in a site-specific manner, with a high preference for polycomb target sites [10]. In keeping with this, our studies show that over 70% of the excess methylated sites associated with in vitro differentiated mouse ES cells are also marked with above-background levels of H3K27me3 (P<10^−159^) in the parent line. Furthermore, we have observed that the degree of methylation following differentiation is linearly proportional to the underlying pre-set local concentration of this histone modification in undifferentiated ES cells as determined from several different studies (Fig. 3a & b). This suggests that the polycomb system may play a role in this targeting process and this is consistent with the finding that Ezh2, a member of the polycomb complex, is actually capable of directly recruiting DNA methyl transferases [33], [34].

While de novo methylation may indeed be directed by polycomb marking in undifferentiated cells, once it occurs, the presence of methyl groups appears to bring about a dramatic reduction in H3K27me3 levels at these same sites as measured in the resulting differentiated cells (Fig. S3 in File S1). This reciprocal effect between DNA and histone modification has also been noted for the de novo methylation that occurs in cancer [35], [36]. It thus appears that during in vitro differentiation many sites of polycomb repression are getting replaced with more permanent DNA methylation silencing, thus, perhaps, limiting regulatory plasticity.

The primary bimodal methylation pattern of the genome is formed in vivo at the time of implantation by a combination of global de novo methylation together with CpG island protection, and ES cells represent a good model for this process. Although these unmethylated windows are ultimately dictated by underlying sequence information, ChIP analysis in ES cells indicates that the presence of H3K4me3 may take part in this protection, since this modification is associated with every unmethylated island and is almost completely absent from those regions that undergo de novo methylation [10]. In order to test whether H3K4me3 may also play a role in modulating the abnormal de novo methylation that takes place during ES-cell differentiation in vitro, we next analyzed the degree of this modification as a function of H3K4 methylation (Fig. 3c). These results clearly show that while sites of polycomb are all targets for this reaction, the co-presence of high-level H3K4me3 at these same CpG islands appears to have a concentration-dependent protective effect.

In general, there appear to be two modes of de novo methylation in vivo, global as opposed to targeted [4]. While aberrant methylation in mouse ES cells is correlated with polycomb targeting (Fig. 3), this does not seem to be true of the abnormal methylation observed in human ES cells, which shows only a marginal enrichment for CpG islands that bind the polycomb complex (Fig. S4a in File S1). One possibility is that in these cells, excess modification comes about by a distortion of the implantation-specific global DNA methylation that operates constitutively in dividing ES cells. As previously noted, this global process brings about non-specific methylation of the entire genome, while sparing CpG islands, and this protection has been shown to be highly correlated with the level of H3K4me3 in local nucleosomes [10].

In order to determine whether this underlying process may be disrupted in human ES cells, we further analyzed histone modification patterns of the aberrantly methylated CpG islands. Despite the lack of enrichment for polycomb, these islands are characterized by a preferentially low spectrum of H3K4me3, both as measured in ES cells themselves as well as other tissues (Fig. S4b in File S1). Previous studies have shown that it is possible to derive an accurate algorithm for predicting which DNA sequences are protected from de novo methylation at the time of implantation [10]. Strikingly, our analysis indicates that the same sites abnormally modified in human ES cells are characterized by a relatively low algorithm score for their potential to remain unmethylated (Fig. S4a in File S1). Taken together, these results are consistent with the idea that these sites are indeed the least capable of being protected from global methylation.

**Figure 4:**
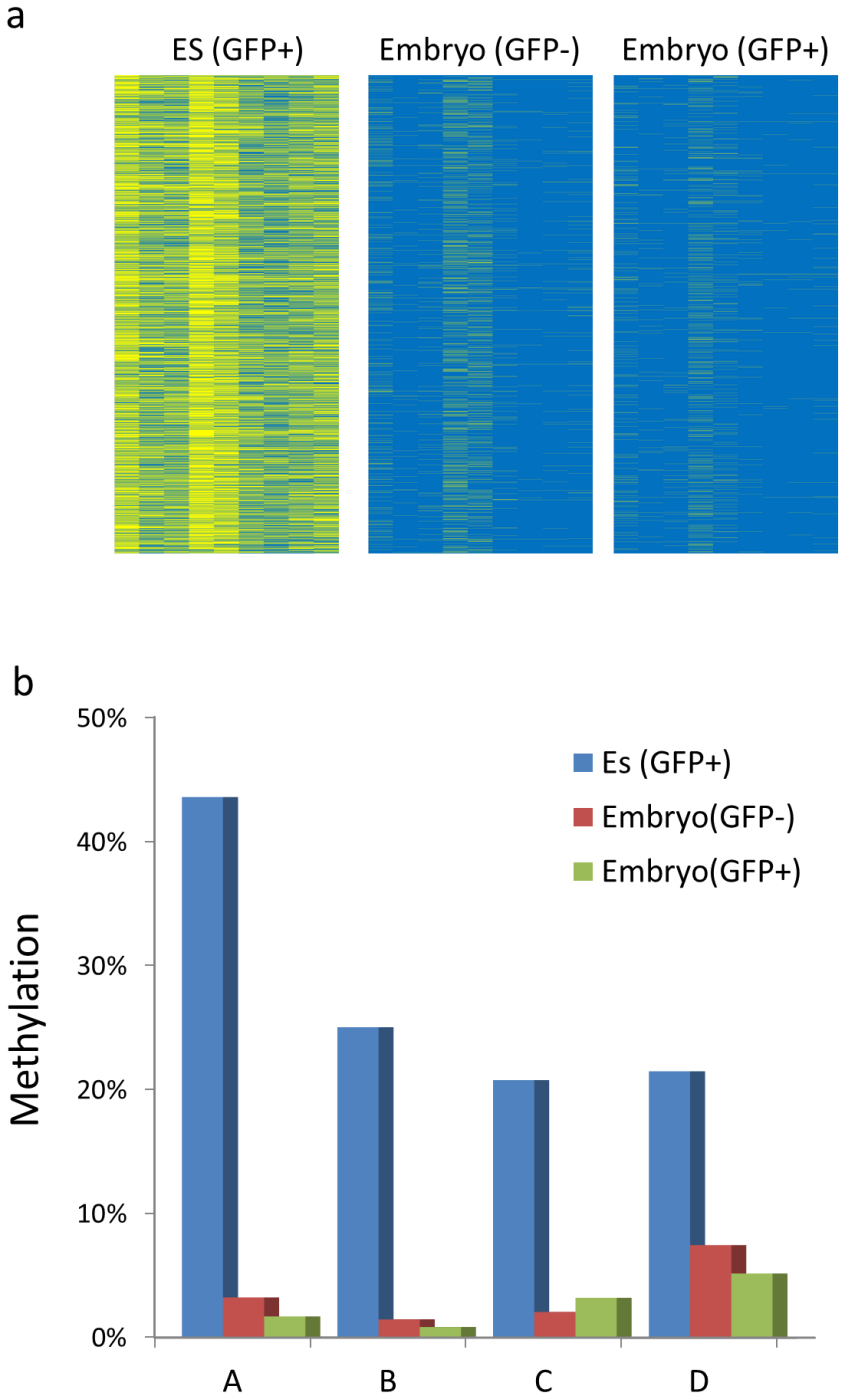
Resetting de novo methylation in vivo. Blastocysts injected with ES cells carrying a GFP expression vector were transplanted into pseudo-pregnant mice. Whole embryos were isolated at 16 dpc and sorted for GFP^+^ and GFP^−^ cells. DNA from these cells was then treated with bisulfite and deep-sequenced (Ion Torrent) at four different specific CpG island sequences. **a**. 7,000 individual molecules of island A containing nine individual CpGs with yellow indicating methylation. **b**. Graph showing percent methylation for islands A, B, C and D.

It is well known that ES cells grown in culture can fully contribute to normal development when incorporated into blastocysts and transplanted into pseudo-pregnant females. In light of the findings that these cells have abnormal methylation patterns to begin with, we next asked whether this modification actually persists during normal development. To this end, we took advantage of an ES cell line that expresses GFP, generated chimeric embryos by blastocyst injection and then separated out the labelled somatic cells from 17.5 dpc embryos using FACS. In the original ES cell line, we were able to detect heavy abnormal methylation in many sample CpG islands. In contrast, the in vivo-derived tissues from labelled cells were completely lacking this mark at all of the tested CpG islands and thus had a pattern similar to normal embryonic DNA (Fig. 4).

This experiment suggests that during normal development early embryonic cells have an inherent ability to remove aberrantly modified sequences. This process could be carried out by a CpG island-specific demethylation pathway similar to that which occurs at select loci in the ICM at the time of implantation [5] and which has been previously identified in ES cells [7], [37], [38]. Alternatively, it may come about because the early embryonic environment induces global demethylation [5], thereby clearing the slate prior to the de novo methylation which would ensue upon implantation. A similar phenomenon is observed when ES cells undergo differentiation during the formation of teratomas in vivo. In this case as well, sites abnormally modified in tissue culture underwent partial demethylation when placed in an in vivo setting (Fig. 1). It should be noted that cell selection could also operate to reset aberrant levels of DNA methylation accrued by prolonged culture.

Even though the de novo methylation that takes place in vitro appears to be aberrant in terms of normal development, one is struck by the fact that this process is clearly programmed at the level of protein–DNA interactions. It thus appears that the logic and apparatus used to carry out this targeted methylation is built into the genome. For this reason, we asked whether there may be instances where this same form of modification actually occurs in vivo. Since this was not observed in normal embryos or somatic tissues, we next examined extraembryonic tissues, such as placenta. Strikingly, mouse placenta at both 14.5 and 19.5 dpc was found to have modified CpG islands with a high percentage (80%) of them representing polycomb targets (P<10^−229^) (Fig. S5 in File S1), as has been noted previously [39]. Similar results were obtained in human placenta, as well. These experiments suggest that CpG island methylation of the type seen in differentiated cells in culture actually mimics a process of modification that takes place during normal extraembryonic development in vivo.

## Discussion

By means of a tracing method that uses data from adult and fetal tissues to determine the CpG island methylation pattern of early embryonic cells, we have been able to define a common baseline for DNA methylation. With this as a standard for the first stages of methylation at the time of implantation, we have found that both mouse and human embryonic stem cell lines carry an aberrant pattern of excess methylation that is made worse by a variety of in vitro differentiation strategies. This effect clearly represents an artifact of growing cells in culture, firstly, because levels of CpG island methylation in normal differentiated tissues are much lower, and because abnormal modification does not occur when ES cells are grown into teratomas or introduced into the normal blastocyst, thereby undergoing differentiation in an in vivo setting. It is not known whether these defects in methylation represent an intrinsic aspect of growth in culture per se, or whether it may be possible to create conditions in vitro that might prevent the appearance of this phenomenon.

Any attempt to understand the molecular mechanism of abnormal methylation must take into consideration the parameters that control methyl group metabolism in ES cells. Even though mouse ES cells are initially derived from the ICM at a stage where most methyl groups are erased from the genome [5], they appear to have a fully developed bimodal pattern of methylation whereby most of the genome is highly methylated, with CpG islands remaining unmodified. This suggests that from the point of view of methylation, these in vitro cells behave as if they are actually at a slightly more advanced stage of development, one that perhaps mimics the early implantation embryo. In keeping with this, it has been shown that these cells actively de novo methylate newly-introduced DNA and also have the capacity to protect CpG island sequences from this process [6], [7]. These properties are probably unique to embryonic stem cells, as it has been shown both in vivo and in vitro that DNA inserted into somatic cells does not generally become de novo methylated [8], [40].

During the very early stages of post-implantation development in vivo, pluripotent cells lose their ability to constantly set up and reset global DNA methylation, but the basic overall pattern initially formed by this process is retained through all subsequent cell divisions by a semi-conservative maintenance mechanism [4]. Although it is not known exactly when this transition actually occurs, it is clear that it must involve downregulation of de novo methylation activity [41] as well as a shift from global to locus-specific recruitment [17]. In addition, differentiation may bring about changes in the protective ability of specific CpG island sequences. Our studies suggest that the growth of ES cells and their differentiation in vitro may upset this delicate balance, and as a result we observe aberrant methylation on CpG islands that are normally preserved in an unmethylated form in vivo. This overmethylation appears to take place on two different levels, global and locus-specific.

The dominant de novo methylation in mouse ES cells is directed to CpG islands that are targets for polycomb. It has been known for a long time that differentiated somatic cells grown for extended periods of time in tissue culture also acquire an aberrant methylation pattern [42]. To emphasize this point, we used our tracing approach to analyze the methylation patterns of long-standing immortalized α and β pancreatic islet cell lines and this revealed over 1,200 aberrantly methylated regions as compared to normal β cells purified directly from fresh pancreatic tissue (P<10^−96^) (Fig. S6 in File S1). Here too, over 80% of the methylated sites appear to be polycomb targets, and the same is true for the hypermethylation observed in cancer [43], [44], [45]. Our studies in ES cells clearly show that the degree of abnormal methylation is linearly dependent on the concentration of H3K27me3 present at these loci prior to differentiation (Fig. 3), and it is thus very likely that this repression complex, itself, actually mediates the biochemical recruitment of Dnmts to these sites [33], [34].

It appears that in vivo, the levels of recruited de novo methylases are very low [45], and as a result, almost all polycomb targets remain relatively unmethylated, showing only a slow increase as a function of aging [46], [47], [48]. However, under stress conditions in culture or during tumorigenesis, there may be increased cellular levels of Dnmt3a and 3b [49], [50] that would then lead to the appearance of abnormal methylation. Undifferentiated mouse ES cells in culture which mimic the implantation stage of development, contain high levels of non-targeted Dnmt3a/3b [41]. It is possible that differentiation in vitro brings about abnormal changes in the distribution of these enzymes, thus causing polycomb targets to undergo aberrant modification.

In human ES cells, a great deal of abnormal methylation is already apparent in the undifferentiated state, and bioinformatics analysis indicates that this does not preferentially occur at polycomb targets. Rather, we observe de novo methylation over a wide range of CpG islands, with preference for sequences having a borderline inherent ability to be protected from this process. This suggests that the aberrant modification observed in human ES cells comes about through a distortion in the general process of global methylation. In vivo, general de novo methylation presumably takes place over a very short window of time. In contrast, cells growing in culture are constantly exposed to the methylation machinery over many generations, and this may bring about an imbalance in the quantitative relationship between the level of de novo methylation and the factors involved in keeping CpG islands unmodified, thereby bringing about a shift in the threshold for protection.

This could take place preferentially in human ES cells because they are regarded as more comparable to mouse Epi stem cells, a developmentally more advanced cell population [51]. It should be noted that this abnormal modification would not have been detected without comparison to the pattern determined by the developmental tracing strategy adopted in our laboratory. Furthermore, studies comparing somatic cell-derived iPS to embryo-derived ES cells [52] are also flawed in this regard, failing to take into consideration the possibility that the pattern in ES cells may be abnormal to begin with (Fig. S2 in File S1). Despite differences in the abnormal methylation observed in mouse vs. human ES ells, it should be noted that the patterns themselves are generated by a common mechanism that involves protection by H3K4me3 regardless of the source of de novo methylation.

We have documented a considerable number of CpG islands that get abnormally methylated in mouse ES cells, and a very large percentage of these are located at promoters of genes involved in differentiation or development (Table S1 in File S1). It is thus very likely that this modification has profound effects on both cell function and developmental plasticity. The precise influence of abnormal DNA methylation is difficult to evaluate due to the lack of expression profiles for equivalent normal cells in vivo. In general, however, almost all of the genes that undergo de novo methylation during the differentiation of mouse ES cells have low expression levels in the undifferentiated cells [13], [53], and this is consistent with the idea that a large percentage of these sites, while not DNA methylated, have bivalent promoters that are repressed by the polycomb complex. These same genes, however, represent important components required for differentiation, and if they become methylated in culture, this could strongly affect the cells' ability to activate them as part of the normal process of development.

In addition to the developmental problems that may be caused by DNA methylation, it should be noted that there could also be a safety concern when using human ES cells that will ultimately be transplanted back into patients. Indeed, our analysis indicates that a large percentage of the excess-methylated sites in human ES cells have been found to be hypermethylated in a variety of different tumors (Fig. S7 in File S1). Since these modifications are most likely irreversible in somatic cells, this could readily predispose them to carcinogenesis. Thus, these results should serve as a warning for using current approaches to generate in-vitro differentiated cells for replacement therapy. Our molecular studies will hopefully pave the way towards deciphering the mechanism of this abnormal process and finding strategies for correcting it.

Abnormal hypo- and hyper-methylation of CpG islands is observed in a wide variety of tumors, in somatic cells grown in culture and following differentiation of embryonic stem cells in vitro [54]. In most of these cases it has been shown that de novo modification is specifically directed to polycomb targets. This suggests the existence of an inherent process that is programmed at the genome level, and raises the possibility that this type of targeted de novo methylation may also occur during normal development in vivo. In this paper, we demonstrate that a similar epigenetic pathway is indeed associated with de novo methylation in both mouse and human placenta [39], [55], [56], [57]. Previous studies already showed that extraembryonic tissues also undergo generalized hypo-methylation [58]. While its role has not yet been elucidated, it is very likely that this modification program has some influence on placental physiology. A similar type of de novo methylation also accumulates in many cell types during the normal process of aging [46], [47], [48]. In this sense, the abnormal modification seen in tissue culture, while triggered by local environmental conditions, may actually be mediated by an inherent regulatory program.

## Supporting Information

File S1 **Supporting Information.** **Figure S1.** Bisulfite methylation analysis of mouse ES cells. **a**. Heat map of RRBS methylation data for normal mouse tissues, undifferentiated and differentiated ES cells (NPC) and implantation embryos showing the top 2,000 CpG islands found modified (>25%) in NPCs. It should be noted that 70% of the aberrantly-methylated CpG islands detailed in Fig. 1 overlap with the set shown here. **b**. Methylation distribution in undifferentiated ES cells for 9,500 constitutively unmethylated (gray) CpG islands and the 2,000 CpG islands shown in **a** that were found to be methylated in NPCs (blue). **c**. Methylation distribution (IMS, derived from Fig. 1) in undifferentiated ES cells for the 9,500 constitutively unmethylated CpG islands (gray) as opposed to the 1,000 CpG islands (green) methylated following in vitro differentiation to endoderm. **Figure S2**. DNA methylation in human ES cells. Heat map analysis of CpG islands in human ES cells and normal tissues as determined by RRBS (Road map). Group A includes CpG islands that are abnormally methylated (>60%) in at least one undifferentiated or differentiated ES cell type but constitutively unmethylated (<20%) in all normal adult and embryo tissues. Group B includes CpG islands that are methylated in a single tissue type but unmethylated in others. For both groups, the same CpG islands appear to be methylated in iPS cells, as well. **Figure S3**. Scatter plot of H3K27me3 density in NPCs vs. undifferentiated mouse ES cells for 9,500 CpG islands (see Fig. S1). The 2,000 aberrantly methylated islands (marked in red) show a dramatic reduction in H3K27me3 density when ES cells are converted to NPCs (a). H3K27me3 density in mouse brain is shown for comparison (b). **Figure S4**. Markers of de novo methylation. **a**. Table showing the percentage of abnormally-methylated CpG islands that are marked with polycomb (H3K27me3>2) and their average algorithm score (A2) both in human and mouse ES cells. IMS data are from Figs. 1 and 2 and methylation sequencing data are from Figs. S1 and S2. The intrinsic ability of any CpG island to protect against de novo methylation can be expressed as an algorithm that takes into consideration underlying sequence features. Islands that are constitutively unmethylated have a high score (average of 2.0), while methylated islands have a low score (−1.80). Differentiated mouse ES-cell methylation targets (Fig. 1) have, on average, an intermediate score (0.8) that is significantly different (P<10^−63^) than the constitutively unmethylated CpG islands. **b**. Methylation levels of all CpG islands as a function of local H3K4me3 density for human ES cells before and after differentiation to endoderm. The average levels in a collection of fetal tissues is shown for comparison. **Figure S5**. DNA methylation in placenta. DNA from normal mouse embryos and from placenta were subject to mDIP microarray analysis. Heat map of 1,000 CpG islands methylated in placenta out of 9,500 background CpG islands (see Fig. 1). An estimate for the average percent methylation is also shown. **Figure S6**. DNA methylation in mouse pancreatic α and β cells. DNA from mouse pancreatic cell lines (TC and Min6) and natural β cells purified form fresh pancreatic islets were subject to mDIP microarray analysis. Out of 9,500 background CpG islands (Fig. 1), IMS Heat map of 2,000 CpG islands deemed methylated (IMS>0.75) in either the α or β cell lines as compared to DNA from embryos (where none have a positive binary methylation score). Only 5 are actually methylated in ex-vivo β cells. An estimate for the average percent methylation is also shown. Ex-vivo cells were obtained by preparing pancreatic islets from 2 to 8 month old transgenic mice carrying a Pdx1-GFP construct by ductal perfusion with collagenase. Islets were then hand-picked and dissociated to single cells with trypsin and subjected to FAC sorting using Anti-insulin antibodies. **Figure S7**. Methylation levels in cancer. Heat map of CpG islands deemed methylated in human ES cells (Fig. 2) compared to methylation levels (P value) in a number of different tumor samples from the Cancer Genome Atlas as determined by Infinium Human Methylation 450 array assay and methylation levels (%) in normal tissues as determined by RRBS from the Roadmap Epigenomics Project (www.roadmapepignenomics.org). The excess methylation seen in each tumor type is significantly greater than that observed in normal tissues (minimal P value<10^−80^). **Table S1**. Genes involved in differentiation and development that are associated with aberrantly methylated CpG islands.
(DOC)
