# Supplementary material for: Aberrant DNA Methylation in ES Cells
Source: PLoS One. 2014 May 22;9(5):e96090. doi: 10.1371/journal.pone.0096090 (PMC4031077; doi:10.1371/journal.pone.0096090)

**Aberrant DNA methylation in ES cells**

Guy Ludwig^1🛆^, Deborah Nejman^1🛆^, Merav Hecht^1^, Shari Orlanski^1^, Monther Abu-Remaileh^1^, Ofra Yanuka^2^, Oded Sandler^3^, Amichai Marx^3^, Douglas Roberts^4^, Nissim Benvenisty^2^, Yehudit Bergman^1^, Monica Mendelsohn^5^ and Howard Cedar^1Ù^

^1^ Department of Developmental Biology and Cancer Research,

The Hebrew University–Hadassah Medical School, Jerusalem, Israel

^2^ Department of Genetics, Silberman Institute of Life Sciences,

The Hebrew University, Jerusalem, Israel

^3^ Department of Biochemistry and Molecular Biology,

The Hebrew University–Hadassah Medical School, Jerusalem, Israel

^4^ Agilent Technologies, Inc., Agilent Laboratories, Santa Clara, California, U.S.A.

^5^ Department of Biochemistry and Molecular Biophysics, Columbia College of Physicians and Surgeons, New York, N.Y., U.S.A.

**Contents**

The file contains Supplementary Figures S1 to S7, and Supplementary Table S1.


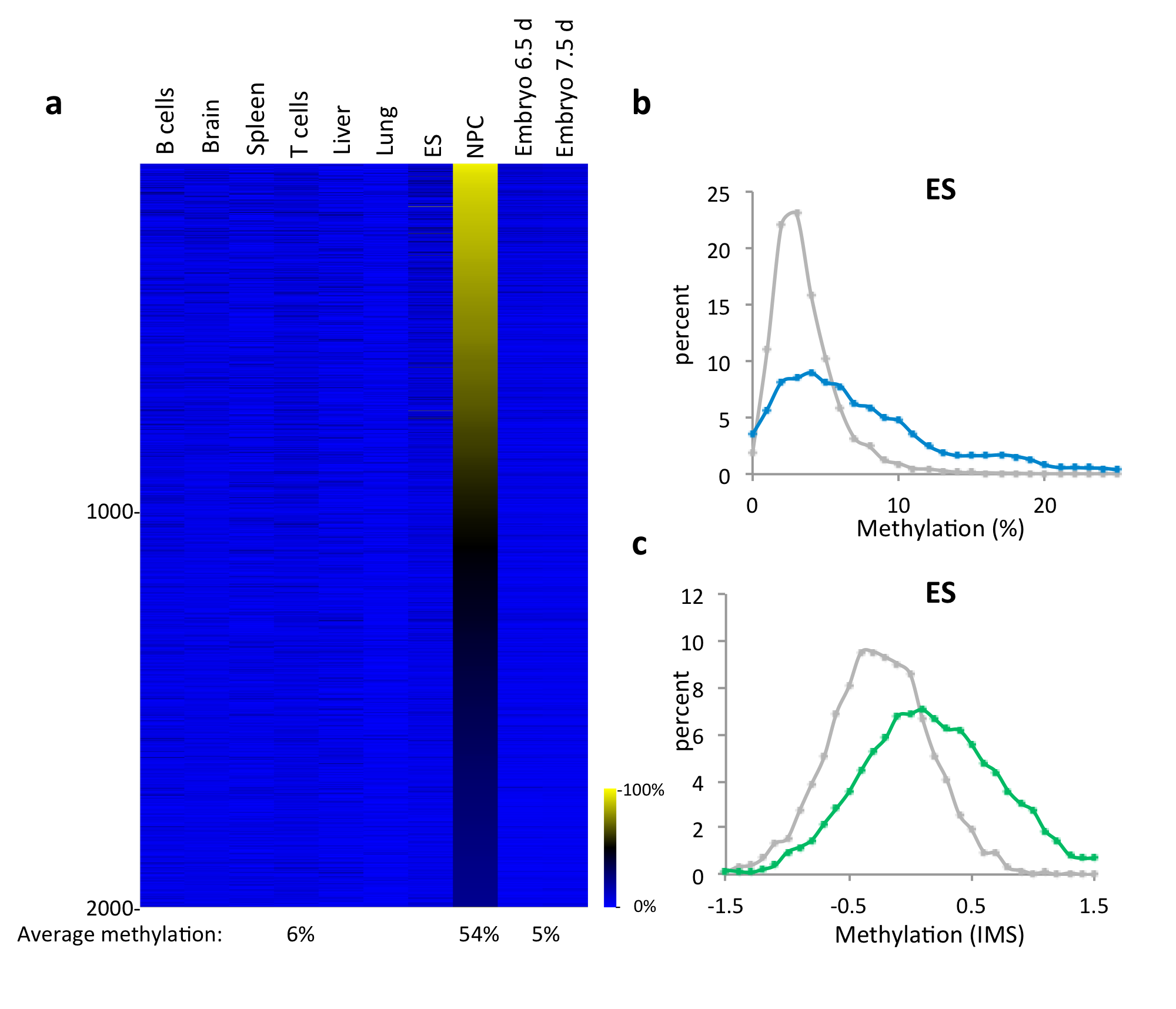
**Supplementary Figures**

**Figure S1. Bisulfite methylation analysis of mouse ES cells.**

**a.** Heat map of RRBS methylation data for normal mouse tissues, undifferentiated and differentiated ES cells (NPC)^1^ and implantation embryos^2^ showing the top 2,000 CpG islands found modified (> 25%) in NPCs. It should be noted that 70% of the aberrantly-methylated CpG islands detailed in Fig. 1 overlap with the set shown here. **b.**Methylation distribution in undifferentiated ES cells for 9,500 constitutively unmethylated (gray) CpG islands and the 2,000 CpG islands shown in **a** that were found to be methylated in NPCs (blue). **c.**Methylation distribution (IMS, derived from Fig. 1) in undifferentiated ES cells for the 9,500 constitutively unmethylated CpG islands (gray) as opposed to the 1,000 CpG islands (green) methylated following in vitro differentiation to endoderm.

**References**

1. Meissner, A. *et al.* Genome-scale DNA methylation maps of pluripotent and differentiated cells. *Nature* **454**, 766-770 (2008)

2. Smith, Z.D. *et al.* A unique regulatory phase of DNA methylation in the early mammalian embryo. *Nature* **484**, 339-344 (2012)

**
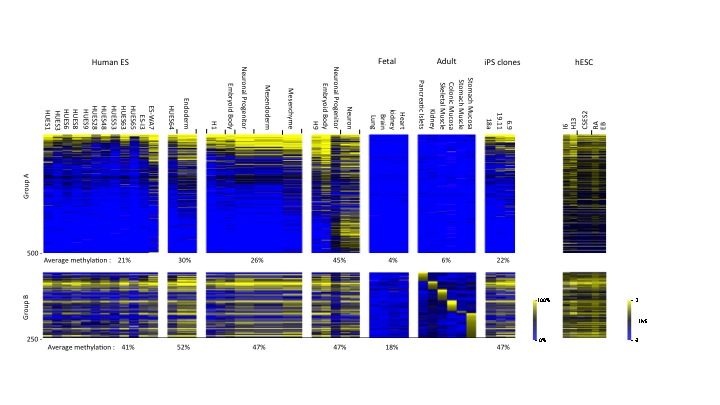
**

**Figure S2. DNA methylation in human ES cells.**

Heat map analysis of CpG islands in human ES cells and normal tissues as determined by RRBS (Road map). Group A includes CpG islands that are abnormally methylated (> 60%) in at least one undifferentiated or differentiated ES cell type but constitutively unmethylated (< 20%) in all normal adult and embryo tissues. Group B includes CpG islands that are methylated in a single tissue type but unmethylated in others. For both groups, the same CpG islands appear to be methylated in iPS cells, as well.

**
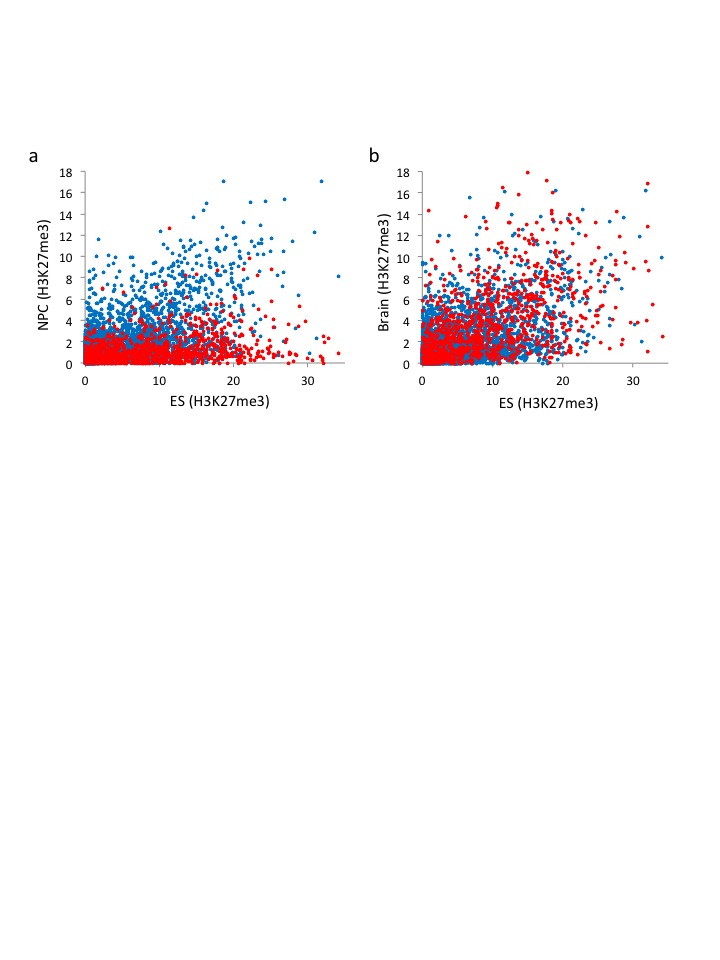
**

**Figure S3**

Scatter plot of H3K27me3 density in NPCs vs. undifferentiated mouse ES cells for 9,500 CpG islands (see Supplementary Fig. 1). The 2,000 aberrantly methylated islands (marked in red) show a dramatic reduction in H3K27me3 density when ES cells are converted to NPCs (a). H3K27me3 density in mouse brain is shown for comparison (b).


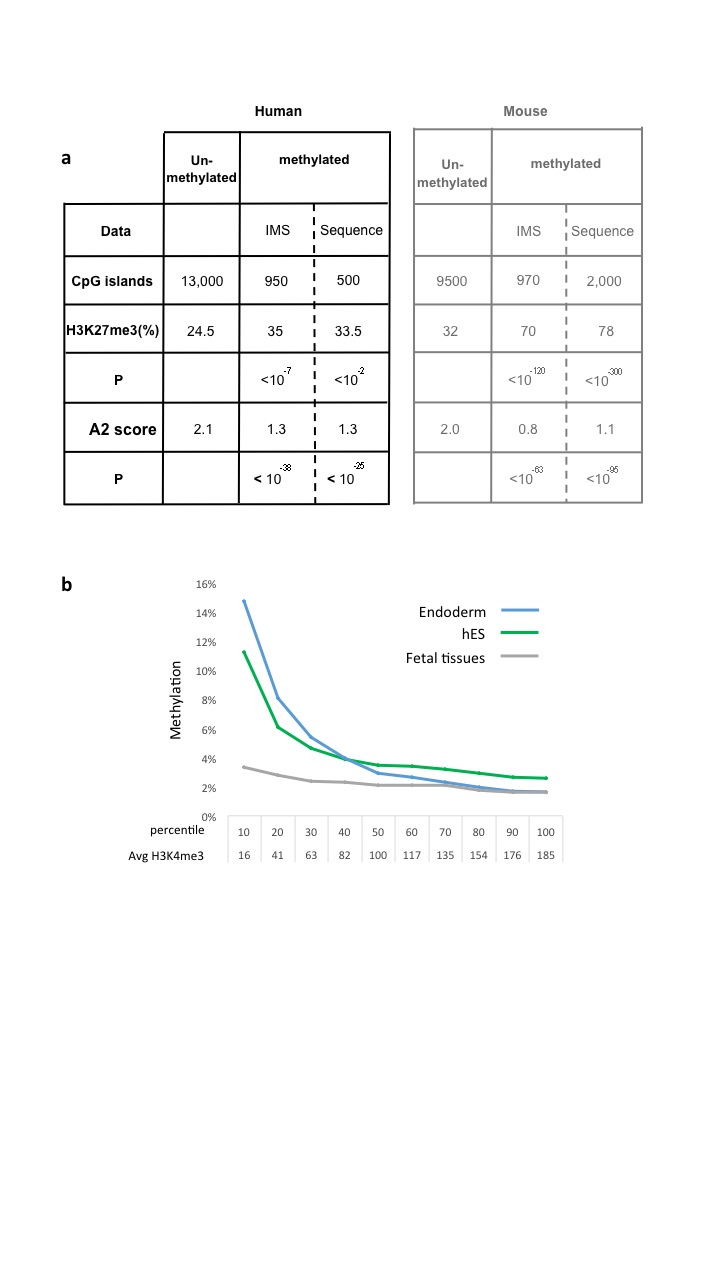
**Figure S4. Markers of de novo methylation.**

**a.**Table showing the percentage of abnormally-methylated CpG islands that are marked with polycomb (H3K27me3 > 2) and their average algorithm score (A2) both in human and mouse ES cells. IMS data are from Figs. 1 and 2 and methylation sequencing data are from Supplementary Figs. 1 and 2. The intrinsic ability of any CpG island to protect against de novo methylation can be expressed as an algorithm that takes into consideration underlying sequence features[^1^](#_ENREF_7). Islands that are constitutively unmethylated have a high score (average of 2.0), while methylated islands have a low score (–1.80). Differentiated mouse ES-cell methylation targets (Fig. 1) have, on average, an intermediate score (0.8) that is significantly different (P < 10^-63^) than the constitutively unmethylated CpG islands. **b.** Methylation levels of all CpG islands as a function of local H3K4me3 density for human ES cells before and after differentiation to endoderm. The average levels in a collection of fetal tissues is shown for comparison.

**Reference**

1. Straussman, R. *et al.* Developmental programming of CpG island methylation profiles in the human genome. *Nature Struct. Mol. Biol.* **16**, 564-571 (2009)

**
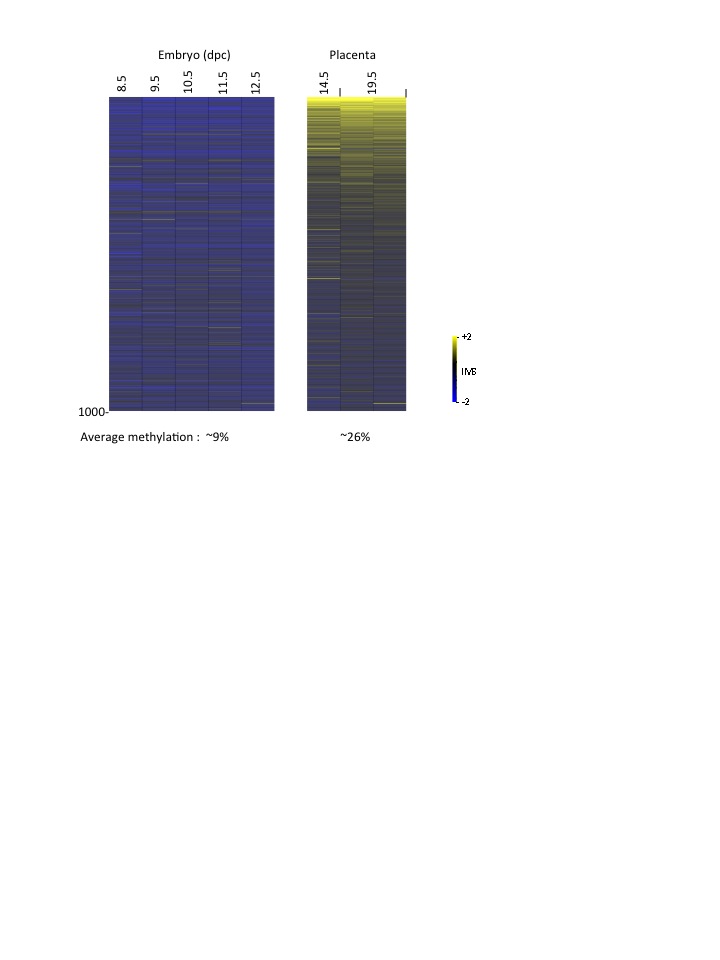
**

**Figure S5. DNA methylation in placenta.**

DNA from normal mouse embryos and from placenta were subject to mDIP microarray analysis. Heat map of 1,000 CpG islands methylated in placenta out of 9,500 background CpG islands (see Fig. 1). An estimate for the average percent methylation is also shown.


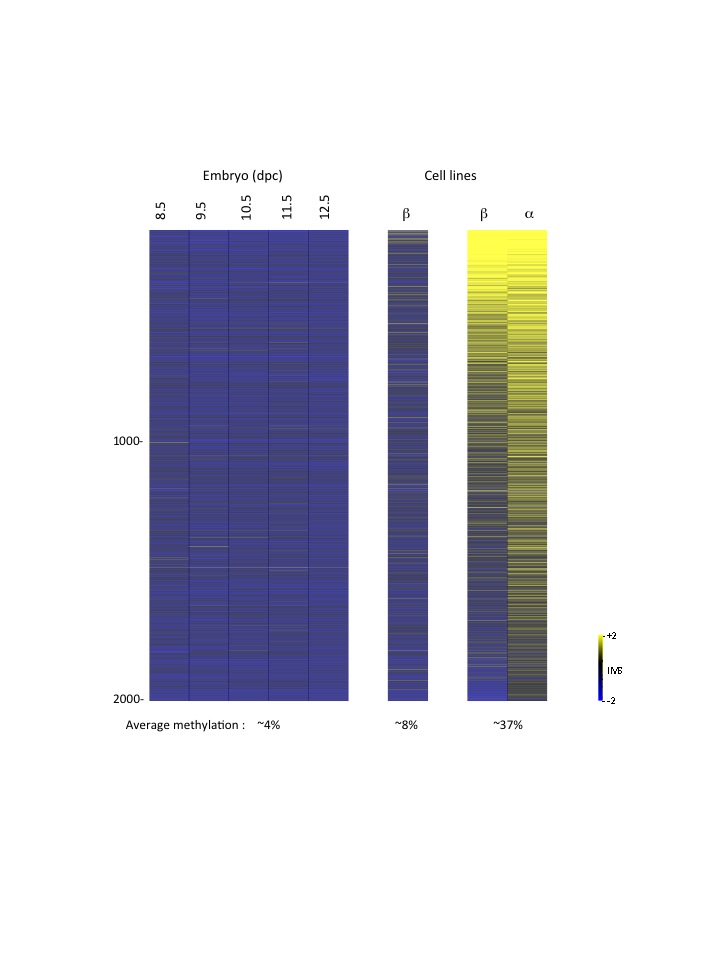
**Figure S6. DNA methylation in mouse pancreatic α** **and β** **cells.**

DNA from mouse pancreatic cell lines (TC and Min6) and natural β cells purified form fresh pancreatic islets were subject to mDIP microarray analysis. Out of 9,500 background CpG islands (Fig. 1), IMS Heat map of 2,000 CpG islands deemed methylated (IMS > 0.75) in either the α or β cell lines as compared to DNA from embryos (where none have a positive binary methylation score). Only 5 are actually methylated in ex-vivo β cells. An estimate for the average percent methylation is also shown.

Ex-vivo cells were obtained by preparing pancreatic islets from 2 to 8 month old transgenic mice carrying a Pdx1-GFP construct^1^ by ductal perfusion with collagenase. Islets were then hand-picked and dissociated to single cells with trypsin and subjected to FAC sorting using Anti-insulin antibodies.

**Reference**

1. Micallef, S.J. *et al.* Retinoic acid induces Pdx1-positive endoderm in differentiating mouse embryonic stem cells. *Diabetes* **54**, 301-305 (2005)

**
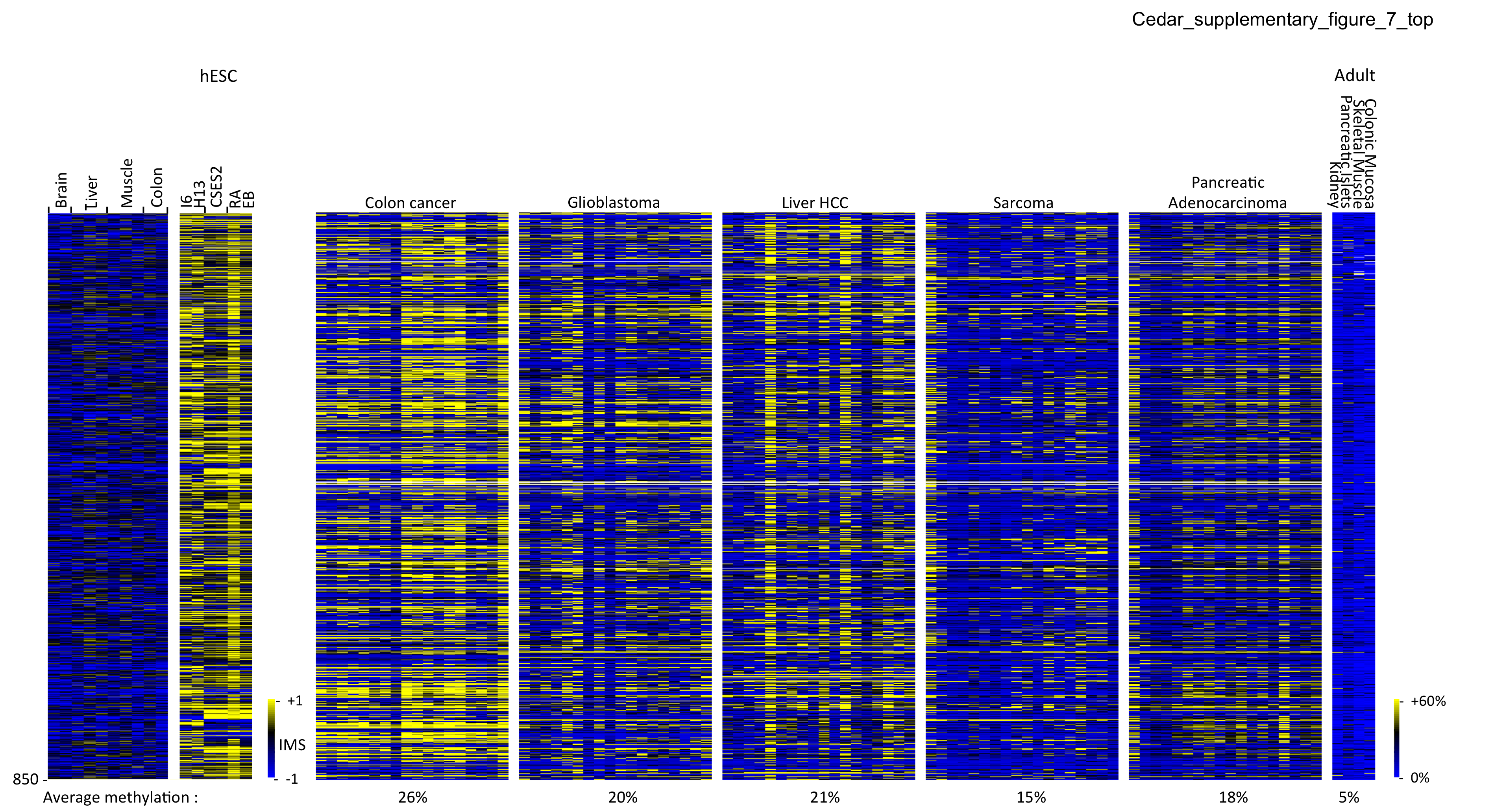
**

**Figure S7. Methylation levels in cancer.**

Heat map of CpG islands deemed methylated in human ES cells (Fig. 2) compared to methylation levels (P value) in a number of different tumor samples from the Cancer Genome Atlas as determined by Infinium Human Methylation 450 array assay and methylation levels (%) in normal tissues as determined by RRBS from the Roadmap Epigenomics Project (www.roadmapepignenomics.org). The excess methylation seen in each tumor type is significantly greater than that observed in normal tissues (minimal P value < 10^-80^).


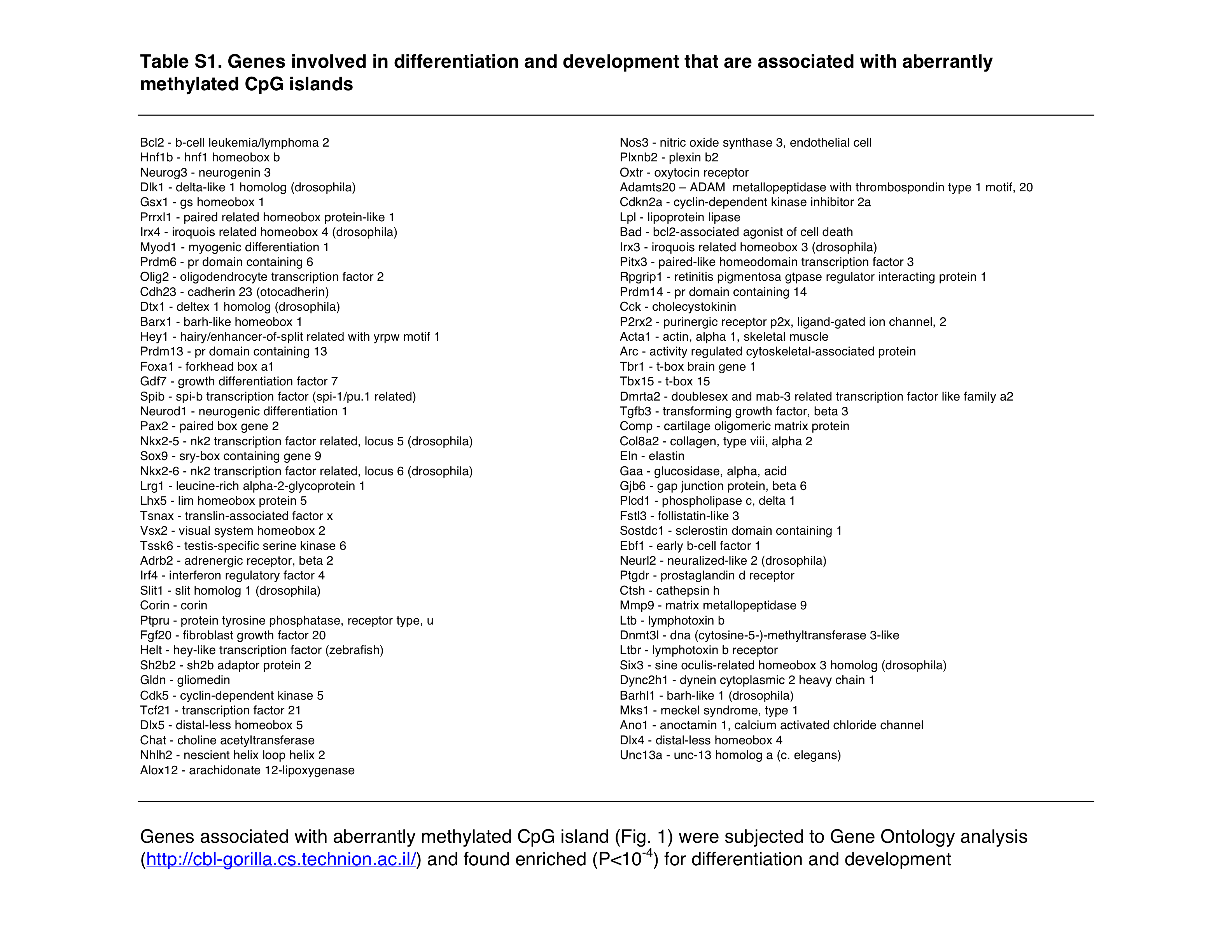

Supplement: File S1 — Supporting Information. Figure S1. Bisulfite methylation analysis of mouse ES cells. a. Heat map of RRBS methylation data for normal mouse tissues, undifferentiated and differentiated ES cells (NPC) and implantation embryos showing the top 2,000 CpG islands found modified (>25%) in NPCs. It should be noted that 70% of the aberrantly-methylated CpG islands detailed in Fig. 1 overlap with the set shown here. b. Methylation distribution in undifferentiated ES cells for 9,500 constitutively unmethylated (gray) CpG islands and the 2,000 CpG islands shown in a that were found to be methylated in NPCs (blue). c. Methylation distribution (IMS, derived from Fig. 1) in undifferentiated ES cells for the 9,500 constitutively unmethylated CpG islands (gray) as opposed to the 1,000 CpG islands (green) methylated following in vitro differentiation to endoderm. Figure S2. DNA methylation in human ES cells. Heat map analysis of CpG islands in human ES cells and normal tissues as determined by RRBS (Road map). Group A includes CpG islands that are abnormally methylated (>60%) in at least one undifferentiated or differentiated ES cell type but constitutively unmethylated (<20%) in all normal adult and embryo tissues. Group B includes CpG islands that are methylated in a single tissue type but unmethylated in others. For both groups, the same CpG islands appear to be methylated in iPS cells, as well. Figure S3. Scatter plot of H3K27me3 density in NPCs vs. undifferentiated mouse ES cells for 9,500 CpG islands (see Fig. S1). The 2,000 aberrantly methylated islands (marked in red) show a dramatic reduction in H3K27me3 density when ES cells are converted to NPCs (a). H3K27me3 density in mouse brain is shown for comparison (b). Figure S4. Markers of de novo methylation. a. Table showing the percentage of abnormally-methylated CpG islands that are marked with polycomb (H3K27me3>2) and their average algorithm score (A2) both in human and mouse ES cells. IMS data are from Figs. 1 and 2 and met [file pone.0096090.s001.doc]
